# Welcome to *AoB PLANTS*: the open access journal for all plant biologists

**DOI:** 10.1093/aobpla/plp001

**Published:** 2009-09-18

**Authors:** Mike Jackson

## Abstract

The Chief Editor of AoB PLANTS highlights some of the key features of this new online, open access journal for plant biologist and sets out some of the reasons for starting.

As the first Chief Editor of *AoB PLANTS* it is a pleasure to introduce this new internationally peer-reviewed, Open Access (OA) journal, owned and run by plant biologists and publishing on all aspects of plant biology. *AoB PLANTS* is being published by Oxford University Press for the Annals of Botany Company, a long-standing not-for-profit organization exclusively concerned with supporting plant biology internationally. *AoB PLANTS* complements the AoB Company's flagship journal *Annals of Botany*. The initial motivation for *AoB PLANTS* was a sustained and sizeable increase in submissions to *Annals of Botany* over a number of years, resulting in the rejection of many valuable and important papers which the journal simply could not accommodate. To cater for the general increase in high-quality manuscripts, *AoB PLANTS* was designed as an online-only journal, not restricted by page extent limitations, and which could use the potential of OA publishing to make its articles available quickly to the widest possible readership. Responses to our recent wide-ranging online questionnaire indicated strong demand for a journal such as *AoB PLANTS*.

## Why open access and online only?

Several difficulties in conventional publishing of peer-reviewed science encouraged *AoB PLANTS* to adopt OA. Open Access helps sidestep library budgets which are struggling to cope with the increasing numbers of journals available, and it makes research freely available to the academic community and beyond. By replacing library subscriptions with modest fees from authors or their supporting institutions, OA is more sustainable than the subscription-based model in an environment where research and paper publication are increasing. This antidote to mounting subscription costs for libraries carries the enormous bonus of giving readers both within and outside the university/research institution system unrestricted access to the most up-to-date information that is available on the internet.

*AoB PLANTS* recognizes that research funds are often hard-won. Accordingly, it will make every effort to minimize the costs to authors. This policy is in keeping with the Journal's not-for-profit ethos. For an initial period, authors will pay no fees, all costs being paid in full by the Journal's owners. In order to remain sustainable, authors will start to be charged at a later stage. However, the cost will increase slowly in several stages but remain highly competitive.

The instant accessibility afforded by OA has been shown to increase PDF downloads in the first 6 months after publication. Thus, readers are more likely to make use of an article if it is published in OA—surely the desired outcome for both authors and their sponsors. In the longer term, citation levels may also benefit from OA. Not surprisingly therefore, OA publishing enjoys the support of numerous large national and international funding organizations. These include: US National Institutes of Health; The Wellcome Trust; Centre National de la Recherche Scientifique (CNRS); UK research councils such as the Biotechnology and Biological Sciences Research Council. These endorsements by major funders of research indicate a move towards OA as the preferred publishing option. They rest on the principle that OA is a way of upholding the public's right to access publicly-funded research without payment and at the earliest opportunity.

Publishing online-only also eliminates the need to reject good manuscripts simply to control the size of the printed journal. Finally, the journal chose to go OA to ensure the widest possible international readership for the growing amount of high-quality plant science being carried out worldwide in response to problems such as climate change and food shortages.

## Defining features of *AoB PLANTS*

There is a plethora of open access journals, including one or two for plant scientists. However, what has been missing until now is a genuinely broad spectrum OA journal managed by plant scientists for plant scientists and one not driven primarily by commercial ambition.

All involved with *AoB PLANTS* intend the new Journal to be a long**-**term success. The journal's management will take a flexible and adaptive approach to publishing *AoB PLANTS* ensuring that it will evolve to satisfy new needs as they arise, exploit new opportunities and deliver content as effectively as possible to the widest virtual community. Many OA journals are highly specialized and cater for narrow subject areas. *AoB PLANTS* will deliver research from all specialties to the entire plant science community.

Short summaries of each paper, the use of structured abstracts of generous length, and the insistence that Introductions to each paper are aimed at non-specialists will appeal to the widest possible range of readers and give maximum exposure to every paper published in *AoB PLANTS*. Publishing authors' manuscripts immediately after acceptance ensures that research is available as soon as possible to readers, before the final version is produced to a very high standard with the help of a sizeable Editorial Board drawn from many countries. I believe these features, coupled to the long-term commitment of an internationally renowned first**-**division publisher (Oxford University Press), and absence of author fees for an introductory period are a winning combination that will attract the confidence of plant biologists worldwide.

